# COVID-19 may exacerbate the clinical, structural and psychological barriers to retention in care among women living with HIV in rural and peri-urban settings in Uganda

**DOI:** 10.1186/s12879-021-06684-6

**Published:** 2021-09-20

**Authors:** Sylivia Nalubega, Joshua Kyenkya, Irene Bagaya, Sylvia Nabukenya, Nelson Ssewankambo, Damalie Nakanjako, Agnes N. Kiragga

**Affiliations:** 1 Department of Nursing, School of Health Sciences, Soroti University, Po Box, 211, Soroti, Uganda; 2 Research Department, Infectious Diseases Institute, College of Health Sciences, Makerere University, Kampala, Uganda; 3 Department of Medicine, School of Medicine, College of Health Sciences, Makerere University, Kampala, Uganda

**Keywords:** Retention, Women, COVID-19, Uganda, Barriers, HIV care

## Abstract

**Background:**

Retention of pregnant and breastfeeding women and their infants in HIV care still remains low in Uganda. Recent literature has shown that the effects of COVID-19 mitigation measures may increase disease burden of common illnesses including HIV, Tuberculosis, Malaria and other key public health outcomes such as maternal mortality. A research program was undertaken to locate disengaged HIV positive women on option B+ and supported them to reengage in care. A 1 year follow up done following the tracing revealed that some women still disengaged from care. We aimed to establish the barriers to and facilitators for reengagement in care among previously traced women on option B+, and how these could have been impacted by the COVID-19 pandemic.

**Methods:**

This was a cross sectional qualitative study using individual interviews conducted in June and July, 2020, a period when the COVID-19 response measures such as lockdown and restrictions on transport were being observed in Uganda. Study participants were drawn from nine peri-urban and rural public healthcare facilities. Purposive sampling was used to select women still engaged in and those who disengaged from care approximately after 1 year since they were last contacted. Seventeen participants were included. Data was analysed using the content analysis approach.

**Results:**

Women reported various barriers that affected their reengagement and retention in care during the COVID-19 pandemic. These included structural barriers such as transport difficulties and financial constraints; clinical barriers which included unsupportive healthcare workers, short supply of drugs, clinic delays, lack of privacy and medicine side effects; and psychosocial barriers such as perceived or experienced stigma and non-disclosure of HIV sero-status. Supportive structures such as family, community-based medicine distribution models, and a friendly healthcare environment were key facilitators to retention in care among this group. The COVID-19 pandemic was reported to exacerbate the barriers to retention in care.

**Conclusions:**

COVID-19 may exacerbate barriers to retention in HIV care among those who have experienced previous disengagement. We recommend community-based models such as drop out centres, peer facilitated distribution and community outreaches as alternative measures for access to ART during the COVID-19 pandemic.

**Supplementary Information:**

The online version contains supplementary material available at 10.1186/s12879-021-06684-6.

## Background

Mother to child transmission of HIV (MTCT) is the most common cause of human immunodeficiency virus (HIV) infection among infants. Despite improvements, Africa still experiences significant new HIV infections among children [1]. Efforts to eliminate MTCT are aimed at early HIV diagnosis and immediate initiation of lifelong antiretroviral therapy (ART) for the women and prophylaxis for all HIV exposed infants up to 18 months (option B+) [2]. The option B+ program provided care services to approximately 82% of all HIV positive pregnant/breastfeeding women in World Health Organisation (WHO) African region in 2018 and has led to an 80% global reduction in MTCT of HIV [1]. Uganda has achieved commendable improvement towards elimination of MTCT of HIV and is earmarked for certification by WHO for pre-elimination status. This status comes with reported increase in ART coverage of 95% among pregnant women in 2018, which resulted into 86% reduction in new HIV infections among children [3]. Despite this achievement, the need to retain the mother-infant pairs in care remains critical in eliminating MTCT [1]. Women are reported to drop out from care across all stages of childbirth following HIV diagnosis, although the post-partum period registers the highest rates of drop outs. In a review done in 2012 by Wettstein, Mugglin [4], drop out of African women from care took a progressive trend in which, of the 94 women who tested HIV positive during antenatal care, 70% initiated ART, 64% of the exposed infants were tested at 6 weeks, while 55% of these children were tested at 18 months. These results, though published earlier remain consistent with more recent reports that have shown an increasing trend of attrition among breastfeeding women [5, 6], indicating retention as a real challenge in achieving elimination of MTCT. More recent data indicates that Uganda’s retention currently stands at 65–75% against the targeted 90% [3].

Various barriers to retention in HIV care and to adherence to HIV medications have been reported, and generally categorised under three types namely; structural (e.g. transport difficulties, accessibility of healthcare facilities and limited finances), clinical (e.g. clinic delays, negative attitudes/experiences with healthcare personnel, clinic delays and fear of drug side effects) and psychosocial (e.g. HIV stigma, disclosure and poor family support) barriers [7]. These barriers tend to be similar across the African region as presented in published literature [7–11]. Similar to the test and treat approach, the option B+ requires women to immediately initiate lifelong ART at diagnosis. These women may be less psychologically prepared for lifelong HIV treatment, may have stigma related challenges, and require to attend a series of healthcare visits which might require more finances. Therefore, retaining pregnant and breastfeeding women in care might require targeted interventions. Because many women may be identified for the first time as HIV infected while pregnant, psychological preparation while initiating ART and during follow up care has been identified as a key need for retaining them in care [12, 13]. Tracing and supporting reengagement in care of previously lost to follow-up clients has also shown to be successful in the general population [9] and could be opted in prevention of mother to child transmission of HIV (PMTCT) services. Other factors documented to facilitate retention in care among pregnant/breastfeeding women include; health benefits of HIV treatment for themselves and their babies, and support from spouses, family and friends [11, 13]. Decentralised community models used in general ART could also be adopted for delivery of PMTCT services to increase retention, however, these are yet to be adopted in Uganda.

Coronavirus disease 2019 (COVID-19) was declared a worldwide pandemic by the WHO on March 11, 2020; and on 21 March 2020, COVID-19 was declared an outbreak in Uganda. The outbreak has since spread to all regions of the country, and by 20th September 2020, Uganda reported over 6000 cases and 63 deaths from COVID-19. In March 2020, measures to control the pandemic were instituted, including lockdown, curfew, social distancing, quarantine, and restriction/cessation of public and private transport. These measures were lifted in a phased manner beginning from August 2020 and by March 2021, the country had relatively resumed normal activities with minimal restrictions especially in the schooling system. Sadly in May 2021, Uganda experienced the second wave of the pandemic which saw many infected, hospitalised and dead. By 19th June, 2020, Uganda had registered 72,679 cumulative cases with 680 cumulative deaths. The second wave prompted reinstating of the COVID-19 response measures, including a total lockdown. The COVID-19 response measures are feared to have a negative impact on access to care for people living with HIV (PLHIV) [14–17]. For example, transport restrictions limit access to healthcare facilities, financial constraints limit affordability of services such as payment for transportation to healthcare facilities, and fear of SARS-CoV-2 infection may hinder people from accessing care at health facilities [14]. In fact, recent literature has shown that the effects of COVID-19 mitigation measures are predicted to increase disease burden of common illnesses including HIV, Tuberculosis, Malaria and other key public health outcomes such as maternal mortality [18]. Among people already at risk of dropping out of care, it is likely that the pandemic will worsen the barriers to retention in care and worsen the previous gains of HIV care programs in Uganda.

A research program was undertaken to locate HIV positive women initiated in HIV care under option B+ and later disengaged from care, to assess their health and wellbeing and that of their infants (LOCATOR STUDY) [19]. Women were considered lost to follow-up if they failed to return for the last three clinic visits, equivalent to 90 days, and had not been identified as having died or transferred out. All disengaged women were counselled and encouraged to reengage into care at their most accessible and convenient health facility. Referral letters to preferred HIV care facilities were provided for women who wanted to re-engage in care. Following tracing and other necessary study procedures, the women were followed up at 1 year to ascertain if they were still retained in care, any challenges that could be affecting their access to care, and their health and wellbeing. The follow up results revealed that some women still disengaged from the care even after community tracing and reengagement in care. In light of COVID-19, we sought to establish whether the COVID-19 pandemic (and associated response measures) has exacerbated the clinical, structural and psychological barriers to retention in care among women living with HIV in rural and peri-urban settings in Uganda, already at risk of dropping out of care.

## Methods

This was a qualitative study, nested in a larger prospective cohort (community tracing) study (LOCATOR) of women who initiated ART for Life during pregnancy as part of the WHO recommended option B+ strategy for prevention of mother to child transmission of HIV [19]. Data were collected from 17 women using in-depth individual interviews, in June and July 2020.

### Study setting and population

The LOCATOR study was conducted in nine public health facilities in Kampala and Wakiso districts in Uganda. The HIV clinics at the facilities were supported by the Infectious Diseases Institute (IDI) as the HIV/AIDS program implementing partner for the Uganda Centers for Disease Control-President’s Emergency Plan for AIDS Relief (CDC-PEPFAR). The study used existing routinely collected data from to identify women initiated in ART retained on care or those who had disengaged from care, based on the information on their clinic encounters. A total of 116 disengaged and 557 retained women were enrolled in the LOCATOR study. The sample included women who initiated antiretroviral therapy, disengaged from HIV care and later traced and reengaged back in care. After one year from the initial community tracing, the disengaged women were interviewed and asked whether they had re-engaged in care or were still disengaged (lost from HIV care). The 1-year follow-up assessment which occurred from April 2020 to July 2020 coincided with the COVID-19 epidemic and the first national lockdown period in Uganda. For this nested qualitative study, a total of 17 women were interviewed to ascertain the impact of the COVID-19 epidemic and associated mitigation measures on existing barriers and facilitators to retention in care.

### Sampling strategy

Both purposive and convenience sampling approaches were utilized to select potential study participants. We aimed to include a maximum of 20 women, with 10 reengaged and 10 disengaged women from the six healthcare facilities. We also aimed to include women in the younger (18–24 years) and older age (25 years and above) brackets. However, three of those contacted (two disengaged and one engaged) did not agree to participate in the in-depth qualitative interviews, hence our sample size included 17 women, with nine engaged in and eight disengaged from care.

### Data collection

Due to COVID-19 response measures that restricted movement, we conducted telephone calls to the participants instead of face to face interviews. This was mainly due to the ongoing restrictions that prohibited the research team from physically meeting the respondents. In-depth individual interviews were conducted using a semi-structured interview guide developed for this study (Additional file 1). Following consent, interviews were recorded using a digital voice recorder. Data collection was conducted in June and July 2020 by two experienced qualitative researchers (main interviewer and assistant).

### Data analysis and maintaining rigor

Recorded interviews were transcribed verbatim and where necessary directly translated before analysis. Data was analysed using the content analysis approach, in which codes, categories and themes were generated. The analysis also drew on the principles of Grounded theory (e.g. constant comparison, theoretical sampling and saturation, and memo writing). Data analysis was done inductively, where codes were generated from the data. A word template was initially used to code the data and following generation of initial codes and tentative categories, data analysis was transferred to NVivo 12 software for further analytical processes, including final categorisation and generation of themes and sub-themes. Attention to rigor was achieved through on-going discussion within the research team regarding each step of the research process. Verbatim quotes were also used to support our interpretations of the data.

## Results

### Characteristics of included participants

Seventeen women, (nine engaged and eight disengaged) participated in the study. More than a half of the participants (59%) were 25 years and above. About a half (53%) of the participants were engaged in care at the time of the interviews. Among the engaged women, only two received their ART from the original health facility of initiation while the rest had transferred to other facilities. Detailed socio demographic data of participants are indicated in Table 1.

**Table 1:** Socio demographic characteristics of participants (n = 17)

Participants ID	Status of care	Current treatment facility	Age range
P1	In care	Same as enrolment	25 and above
P2	In care	Different from enrolment	25 and above
P3	Not in care	NA	25 and above
P4	Not in care	NA	25 and above
P5	In care	Different from enrolment	25 and above
P6	Not in care	NA	18–24
P7	In care	Different from enrolment	25 and above
P8	Not in care	NA	25 and above
P9	In care	Different from enrolment	18–24
P10	Not in care	NA	18–24
P11	Not in care	NA	18–24
P12	In care	Different from enrolment	25 and above
P13	In care	Different from enrolment	18–24
P14	In care	Same as enrolment	25 and above
P15	Not in care	NA	25 and above
P16	Not in care	NA	18–24
P17	In care	Different from enrolment	18–24

### Main study findings

The study aimed to establish the barriers and facilitators for reengagement in care among previously traced women on option B+, and how these factors could have been impacted by the COVID-19 pandemic. The findings are presented under two main themes and several sub-themes, to highlight the barriers to and facilitators of access to HIV care and support for women under option B+. In addition, the findings have been generally presented in light of how the COVID-19 pandemic has influenced the specific barriers/facilitators to access to care by the women. More general barriers and facilitators have also been highlighted.

### Barriers to reengagement and retention in care

Women reported various barriers that affected their reengagement and retention in care, including adherence to HIV medications. These barriers were found to fall under three categories, including; structural, clinical and psychosocial barriers.

### Barriers to access to HIV care in the face of COVID-19

#### Structural barriers

Structural barriers generally refer to the physical, organisational and operational factors that had a negative influence on the ability of women to seek and access healthcare facilities for their treatments [7]. Factors such as transport difficulties (either because facilities are not available or are expensive), and living far from care facilities were key barriers for accessing care during the COVID-19 pandemic). The COVID-19 pandemic was generally reported to affect women’s access to care by relocations to far settings and reduced income through loss of their jobs or that of their care providers such as partners. Additionally, movements were generally affected by the COVID-19 response measures such as restriction of public and private transport which further affected women’s access to care. During the first wave, restrictions on transport were lifted in a phased manner, beginning with private vehicles. Since the majority of the participants were of a low socio-economic status, they relied mainly on motorbike riders (boda-boda) which was among the last to be released to work, making it difficult for them to access healthcare facilities.

*It (COVID-19) affected me so much most especially at the beginning when public transport had just been stopped because I was left with few drugs. It became worse even when the Boda-bodas (motorcycle riders) were stopped from moving. (P13-in care)*

*Transport has really disturbed me so much, even someone who would have given me some money cannot because all they say “I’m no longer working.” (P9-in care)*

Participants felt that authorities would be lenient by allowing easier to access means such as motorbikes (boda-boda) to operate since these are more accessible and affordable to allow access to care during the pandemic.

*They would have stopped the public transport and at least allowed the boda bodas to operate, instead people have to walk to the facilities. (P10-not in care)*

#### Clinical barriers

Clinical barriers generally refer to how the clinical care environment affected access to HIV care and treatment. The COVID-19 pandemic was reported to contribute to the clinical barriers. For example, some women reported that the fear of contracting the Coronavirus demotivated them from going to collect their medications, especially as government directives had strictly recommended social distancing as a mitigation measure:

*Some of us feared to go to the health facilities because we thought there were COVID-19 patients who would infect us. Also when they are health educating at the facilities, over the radios and TVs, they tell us it’s easy for people living with HIV to contract the covid-19 and they are much affected thus discouraging people to go for care. (P12-in care)*

*Yes, it (COVID-19) has affected me because they stopped us from congregating and yet at the health facilities are always crowded. (P8-not in care)*

Taking medication on an empty stomach was another challenge faced by women during the COVID-19 lockdown and it affected adherence to HIV medication. Some women reported having perceived fear of effects of the medications taken on an empty stomach due to lack of food because they could not afford it. One woman who was previously adhering to medication reported how loss of work due to COVID-19 failed her to buy food which affected her adherence to the medications:

*I was working in a Jewellery shop but it was closed ever since COVID-19 started and they haven’t called me back so I’m now planning to get another job. The situation has not been the same because I even tried to take my medication on an empty stomach but I almost died. (P13-in care)*

#### Psychosocial barriers

Psychosocial barriers are those associated with how psychological and social factors affected women’s motivation and ability to access HIV care and/or adhere to HIV medications. These factors were largely associated with the fear of HIV stigma which affected disclosure of the women’s HIV status. The COVID-19 response measures such as lockdown made couples to stay in the same environment longer (e.g. they could no longer go to work) which affected women’s adherence to medication for fear of revealing their HIV status. This was a particular problem for women who had not disclosed to their partners, since they feared their partners could discover their HIV status from the medication they were taking.

*At first I was affected by those measures because my husband was not working but later he went back to work. […] I was not free at home because my husband was always around. […] I used to take it (the medications) from my friend’s place. (P16-not in Care)*

### General barriers to access to HIV care

The study established that although various barriers were related to COVID-19, some were general barriers that existed irrespective of the COVID-19 pandemic. These barriers were reported among the three categories, of structural, clinical, and psychological barriers.

#### Structural barriers

Although problems associated with transport difficulties were more pronounced during the COVID-19 pandemic, some participants reported these as general barriers to access to HIV care. For example, there are participants who were located far from healthcare facilities. Some participants reported relocating to far locations after being initiated in care which interfered with their access to care. Other women generally struggled to raise transport fees due to their poor socio-economic status.

*The major problem now is the distance. The health facility is in Kawanda and yet I no longer stay that side. The other time I was staying in Kawempe which was near and I would some time walk but now it’s hard for me. (P8- not in care)*

*…when I was still staying with my husband before he died, we would have marital misunderstandings and I would end up going to the village of which appointment dates would find me while I’m still there and fail to get transport. (P3-not in care)*

#### Clinical barriers

Clinical barriers were key in women’s choices of a particular healthcare facility and sometimes resulted in drop out from care or relocation from one facility to another. Women in particular reported the negative reactions/treatments from healthcare staff as a major demotivation for their access to care in specific care facilities:

*Unfriendliness of the health care staff as from my previous facility where I was getting treatment from, the medical personnel were backstabbing and really unfriendly to me. This affected me because I could no longer go for my treatment. (P1-in care)*

Other important clinical barriers included being delayed at facilities, facilities with less privacy which appeared to undermine the women’s confidentiality, and facilities which did not offer comprehensive care, but only provided ART or provided medication for shorter periods of time. A specific challenge reported to affect adherence to medication was the fear or actual experience of medication side effects. A woman who reported never initiating her treatment blamed it on the fear of side effects disclosed to her during a healthcare talk of ART initiation:

*The side effects of the drugs that the medical personnel told me were so scaring like dreaming about the snakes, feeling dizzy and fatigued and she said they would persist for over one month. Since I had issues disturbing, I imagined what would have happened after combining the two things. (P6-not in care)*

#### Psychosocial barriers

Generally, the psychosocial barriers reported appeared to centre on HIV related stigma, either internalised or experienced. A number of women reported experiencing negative reactions such as mistreatment and marginalisation from those to whom they disclosed their HIV positive status. Such women regretted taking the decision to disclose while others, for fear of such negative effects refused to disclose. Lack of disclosure was reported to affect seeking care or adherence to medications:

*Let me tell you the truth, to go for medication I just escape from home because I don’t want to tear my family into pieces. (P4-not in care)*

*I used to take my drugs in hiding and I never used to keep it at home but when I disclosed to him, I started taking it freely. (P13-in care)*

Perceptions of being healthy was also a significant barrier for some women to initiate or remain in HIV care. Some felt that HIV treatment was necessary when one had significant health challenges and therefore would be willing to take the medications if/when their health conditions deteriorated. “*I cannot even take the medication while I’m still healthy […] Yes, I’m very fine and healthy so there is no need of taking the medication.” (P15-not in care).*

### Facilitators of re-engagement and retention in care

Our study established various factors that facilitated women to reengage and be retained in care and also adhere to their HIV medications. In the face of the COVID-19 pandemic, participants expressed specific facilitators they thought would enable them to access HIV care. Hence, facilitators have been presented in two parts, first specific facilitators during the COVID-19 pandemic are presented, and after, the general facilitators to access to care are described.

### Facilitators to enable to access to and retention in care during the COVID-19 pandemic

#### Accessible services

A common barrier to access to HIV care during the COVID-19 pandemic was difficulty in accessing transportation to healthcare facilities due to various reasons highlighted earlier. It was reported that having services that are accessible, for example through receiving treatments from nearby facilities or having services brought to communities would facilitate women to remain receiving their HIV treatments. The COVID-19 pandemic which affected the movement and income of women raised a significant need for community-based HIV services.

*According to this situation of Corona disease and which I believe will not end soon, the organisations should establish the number of patients on a certain village and support with delivery of drugs to individuals. They can also take the medication to the village women representatives and they let us know and we pick it from her and we stop moving for long distances because the majority of us do not have transport. (P7-in care)*

During the COVID-19 pandemic, some women reported receiving special support from healthcare workers where their treatments were delivered to their homes, a measure they applauded for facilitating their continuity in care despite the numerous challenges imposed by the pandemic.

*First of all, when I fail to make it to the health facility for my medication, I can ask my counsellor and she brings it for me. Some people don’t have transport during this period (of COVID-19) but for us we were told in case we don’t have transport, we can give1000 shillings to the counsellor and she brings it to us which transport is cheaper compared to the 3000 we always use. (P9-in care)*

Although the community delivery modal was recommended by some participants, it is clear that others were not in support of this approach due to personal reasons, especially HIV associated stigma.

*At the facility, there are other medical personnel coming from villages, one from every village local council (LC) and they are the ones responsible for giving out drugs to the patients and yet they are our family friends so I dare can’t go there. As long as I decide to go there, they will tell everyone. (P4-not in care)*

### General facilitators for access to and retention in care

#### Supportive structures

The support received from various entities such as family members, peers and healthcare workers was essential in enabling women to reengage and remain in care. Participants reported receiving various types of support, including psychosocial and financial/material support. Psychosocial support motivated women to overcome the fear of HIV stigma, while financial support was mainly helpful for facilitating access to care facilities and purchase of food staff which was an important factor in adherence to medication.

*People who have supported me so much are part of my family. My appointment dates are sometimes due when I don’t have transport however my sister has always given me a hand. My mother has been there for me so much. (P7-in care)*

Women recommended the need for psychosocial support by trained counsellors, who are also familiar with them.

*They should do more health education through drama, health education talks through media like over the radios and TVs and they should also give counsellors enough transport to reach more patients because they know them better and patients feel free with them. (P12-in care)*

#### Conducive healthcare facility

Factors associated with healthcare facilities were important in enabling women to remain in care. These included timely services, being attended to privately to avoid disclosure of their HIV status and availability of treatments for various health problems. In addition, caring facility staff who provided psychosocial support and made women feel accepted emerged as an important reason for women to prefer a particular care facility to another.

*The medical workers care for the patients and do not mistreat them but in my former facility while I was still pregnant, I was abused by a medical worker who made me annoyed and I decided to join this current facility. (P17-in care)*

#### Health benefits

Women reported that the desire to be healthy and to have HIV uninfected children highly motivated them to keep in care and adhere to medications. For some, seeing very ill HIV patients was a wakeup call to be serious with their treatments despite other challenges faced.

*The state of my health is very important, I already know I’m HIV positive, I need to encourage myself to go to the health facility and pick my drugs because once I do not pick them, my life can be affected, therefore I need to support my life. (P7-in care)*

*The most important thing (for remaining in care) is my child and also keeping myself healthy. (P9-in care)*

#### HIV status disclosure

Disclosure of HIV status contributed to women’s continued access to care and adherence to medications. Women reported that disclosure of their HIV status to various people removed fear of being stigmatised, hence they were able to seek care and take their medications openly. In addition, disclosure of their HIV status resulted into being supported by those who became aware of their condition, which further motivated their engagement in care.

*I haven’t faced any challenge (since I disclosed) but instead she always calls me to remind me about my appointment dates and I’m very happy for having told her about it. (P17-in care)*

Women reported disclosing to people they considered trusted and likely to be supportive such as their family and friends, and preferred personal to supported disclosure. However, those who found personal disclosure difficult advocated for assisted disclosure.

*…unless if I’m to bring him to you and you help me to disclose to him but personally I can’t because I fear him. (P11-not in care).*

## Discussion

Our study presents findings on how the COVID-19 pandemic has exacerbated the barriers to retention in care among previously disengaged women living with HIV in Uganda, who were initiated in care under the option B+ program. Women expressed varying barriers to access to care which called for several approaches to care delivery. The COVID-19 pandemic was reported to worsen access to healthcare facilities, majorly due to transport difficulties and to fears associated with contracting the coronavirus, if the women mixed in the population. Another key barrier to access to healthcare for the women during the COVID-19 pandemic was the fear of HIV status disclosure (since family members such as spouses lived longer at home). These findings are consistent with recent literature which reports a negative impact of the COVID-19 response measures on access to care for PLHIV [16–18].

It was recommended that community-based services would be appropriate during the COVID-19 pandemic to mitigate most barriers to access to HIV care. Our study revealed that although access to care was more difficult during the COVID-19 pandemic, some women dropped out of care due to difficulties in raising transport costs or living far from healthcare facilities even before the pandemic. Hence, although the community-based approach would be very essential during the pandemic, this could also be maintained afterwards among this group. Community-based models suggested in this study included drop out centres, peer facilitated distribution, and community outreaches. These interventions have been successful in routine ART management among clinically stable patients [20–22] but have not been adopted for pregnant and breastfeeding women on option B+.

However, this research also established that a particular group of participants were not comfortable with the community model of care for fear of disclosure of their HIV positive status. Participants whose access to HIV care was affected by stigma would require other supportive approaches such as transport facilitation to care facilities. Some participants also reported failing to adhere to their HIV medication due to lack of food, and the fear that taking medication on empty stomachs would cause them harm. Food support or cash transfers would be considered as a supportive measure for ART adherence for women who (or whose providers e.g. husbands) stopped earning during the COVID-19 pandemic.

The current study established that there were many other barriers to seeking HIV care among women on option B+ which were unspecific to the COVID-19 pandemic. Many of these were associated with the option B+ model of care, which involved immediate initiation of lifelong HIV treatment upon diagnosis. It was revealed that the fear of stigma and lack of disclosure was among the commonest reason women gave for not going for care as they feared to be identified as HIV positive. Additionally, some women reported refusing treatments since they felt they were not sick at the moment and therefore did not need HIV treatment. Under the option B+, women diagnosed during pregnancy or breastfeeding are immediately initiated on ART irrespective of CD4 count or clinical staging, which makes them likely to miss out on key initiation procedures such as psychological preparation, an essential aspect in retention in care [12, 13]. Women under option B+ are likely not to be well counselled and tend to suffer from stigma related challenges including lack of disclosure [8, 10]. this concern has been reported to be a hindrance to the success of option B+ and the test and treat strategies where, clients initiated under these programs possess a higher possibility of dropping out of care as they perceive themselves as healthy compared with those initiated under the conventional WHO staging criteria [8, 13, 23, 24]. These findings highlight a critical need for adequate preparation of women initiated on ART under option B+. These women also need follow up support to ensure that they have coped with their diagnosis and are ready to independently own their care. Interventions to support disclosure of HIV status, such as couple voluntary counselling and testing, contact tracing and supported disclosure can be an additional facilitator to retention in care for this group [24].

Our study further revealed that many women dropped out of care as a result of a non-favourable care facility. Concerns related with unfriendly staff, clinic delays and lack of privacy at clinics were the major barriers to attending particular care facilities. A review by Camlin and colleagues [25] reported that a major factor for failure to return to clinics among HIV clients was mistreatment by healthcare workers. This and other clinical related challenges have been reported by other authors [7, 26], and contribute greatly to attrition from HIV care. Worse still, women initiated on ART under option B+ may experience these barriers more severely due to the possible lack of psychological preparation [12, 13]. Targeting healthcare workers has been highly recommended [26]. Hence, efforts to intervene at clinical level should also include supporting healthcare workers to deliver the required support for the women according to set regulations.

Finally, this study established that despite tracing and supported reengagement in care, a number of women still disengaged from care. The findings suggested that factors associated with the first disengagement did not significantly differ from those of the second among the disengaged women, even though some were found to be made worse by the COVID-19 pandemic and associated mitigation measures. These findings suggest that while tracing and supporting reengagement in care has been a successful intervention in general ART management [9], it may not be sufficient for women diagnosed and initiated in HIV care during pregnancy. Targeted and person-centred interventions for the reported barriers at the first disengagement [9, 24, 26] is essential in this group. Interventions targeting minimisation of intimate partner violence such as couple voluntary counselling and testing, contact tracing and supported disclosure would be essential since some barriers to access to HIV care relate to intimate partners.

## Limitations

COVID-19 disruptions limited our interaction with participants since we had telephone interviews. Consequently, we experienced some interruptions including poor network connectivity and interrupted concentration from respondents during the telephone interviews. This could have negatively affected the quality of the data that informed this study unlike the face to face interviews. Our study was based on a limited number of women within urban and peri-urban settings in Uganda, who were disengaged from HIV care following initiation on ART under the option B+. These findings may therefore not be generalizable beyond the studied population/context.

## Conclusions

The COVID-19 pandemic is likely to cause further drop out from care among pregnant and breastfeeding women who are already at risk of disengagement from HIV care. Women living with HIV who were diagnosed and initiated on treatment during pregnancy under option B+, need adequate preparation and ongoing psychosocial support to remain in care. Interventions that target couples such as couple voluntary counselling and testing, contact tracing and supported disclosure are critical to enhancing access to care and adherence to medication among women under option B+. We recommend community-based models such as drop out centres, peer facilitated distribution and community outreaches as alternative measures for access to ART during the COVID-19 pandemic, to target women with access to care challenges, while financial/transport facilitation could improve access to care and adherence to medication for the women with psychosocial barriers.

## Supplementary Information

**Additional file 1.** Data collection tool. The general objective of this study is to assess the barriers to and facilitators of re-engagement in care following community tracing among HIV positive women on option B+, in light of the COVID-19 pandemic.

## Data Availability

The datasets used and/or analysed during the current study are available from the corresponding author on reasonable request.

## Abbreviations

AESA: Alliance for Accelerating Excellence in Science in Africa
ART: Antiretroviral therapy
COVID-19: Coronavirus disease 2019
HIV: Human immunodeficiency virus
HRSA: Health Resources and Services Administration
IDI: Infectious Diseases Institute
LC: Local council
MTCT: Mother to child transmission of HIV
PEPFAR: President’s Emergency Plan for AIDS Relief
PLHIV: People living with HIV
PMTCT: Prevention of mother to child transmission of HIV
WHO: World Health Organisation
