# Supplementary material for: COVID-19 may exacerbate the clinical, structural and psychological barriers to retention in care among women living with HIV in rural and peri-urban settings in Uganda
Source: BMC Infect Dis. 2021 Sep 20;21:980. doi: 10.1186/s12879-021-06684-6 (PMC8451386; doi:10.1186/s12879-021-06684-6)
Supplement: Supplementary file 1 — Additional file 1. Data collection tool. The general objective of this study is to assess the barriers to and facilitators of re-engagement in care following community tracing among HIV positive women on option B+, in light of the COVID-19 pandemic. [file 12879_2021_6684_MOESM1_ESM.pdf]

DATA COLLECTION TOOL

The general objective of this study is to assess the barriers to and facilitators of re-engagement in care following community tracing among HIV positive women on option B+, in light of the COVID-19 pandemic.

A. To assess the facilitators for re-engagement in care following community tracing

1. What motivates you to keep in HIV care and receiving your HIV medications?
2. How have you found accessing care at your current facility?
3. What motivates you to continue to access care at your current facility?
4. What other factors/people have been supportive in enabling you to keep accessing HIV care?
5. What else can be done to facilitate you to continue receiving HIV care?

B. To explore the barriers to re-engagement in care following community tracing

1. What are the reasons that have hindered/disrupted you from seeking HIV care?
2. What are the reasons why you would not prefer to access HIV care at your current facility?
3. What other challenges have you encountered during accessing your HIV care?

C. To explore HIV status disclosure challenges and strategies among HIV positive women

1. To whom do you prefer disclosing your HIV status and why?
2. To whom have you disclosed your HIV status?
3. What challenges have you encountered associated with disclosing your HIV status?
4. How has disclosure of your HIV status affected you in terms of seeking HIV care and treatment?
5. What can be done to support disclosure of HIV status among HIV positive women?

D. To assess the impact of Covid-19 on access to care and treatment among HIV positive women

In light of pandemics, there is fear that access to care is affected:

1. How has the Covid-19 situation affected your access to HIV care (e.g. going to hospital, taking medications, etc…)
2. What has changed in terms of access to HIV treatment since the covid-19 breakout
3. What challenges have you encountered as a result of Covid-19 response measures e.g. lockdown, having the spouse at home, stopping public transport, curfew, etc…
4. What has facilitated continuity to HIV care and your ability to take your medications at home? Or despite the Covid-19 situation, what has enabled you to continue accessing care/taking your medications *(especially for those continuing to take medications…)*

E. What do you think should be done (by the government, by healthcare facilities, by NGOs, etc…) to enable women to stay in HIV care
